# Building social capital with elders’ leadership through a community hub “*Ibasho*” in the Philippines and Nepal

**DOI:** 10.1038/s41598-023-30724-7

**Published:** 2023-03-04

**Authors:** Takeshi Aida, Emi Kiyota, Yasuhiro Tanaka, Yasuyuki Sawada

**Affiliations:** 1 Institute of Developing Economies, Japan External Trade Organization (IDE-JETRO), Wakaba 3-2-2, Mihama-ku, Chiba-shi, Chiba 261-8545 Japan; 2 National University of Singapore, Singapore, Singapore; 3 Ibasho Japan, Tokyo, Japan; 4 University of Tokyo, Tokyo, Japan

**Keywords:** Epidemiology, Psychology and behaviour, Health care economics

## Abstract

We quantitatively study the *Ibasho* project—a unique, innovative community-based project that involves co-creating a building as a social hub. *Ibasho*’s decision-making undertakes a bottom-up approach, differentiating itself from the conventional top-down decision-making process. Using sui generis data, we find that *Ibasho* projects in the Philippines and Nepal contributed to enhancing social capital among elders in both cases. Yet there are differences between the two communities. In the Philippines, participation in *Ibasho* increased the number of a participant’s friends, or “strong ties,” indicating that it is on the intensive margin of human relationships. In contrast, joining Nepal’s *Ibasho* broadened weak ties rather than strong ones. This contrast may stem from the difference in pre-existing social and built infrastructures in the two communities, which were strengthened through the building-human interactions.

## Introduction

In almost all parts of the world, the number of elders is growing faster than the total population, challenging the way our societies care for older people and allowing them to remain engaged with purpose^1^. Our current social norms tend to view aging as a process of decline, with the growth that accompanies aging being invisible to societies^2^. As a result, older people are removed from the daily flow of community life and their wisdom and experience are not transferred to future generations. In addition to the loss this represents, isolation puts elders at increased risk of both illness and of vulnerability in the case of a catastrophic event, while also increasing the cost of their care for families and society^3^.

To respond to these challenges, societies worldwide are seeking effective and innovative ways to enable older people to maintain meaningful engagements and remain woven into the fabric of community life. To this end, creating a built environment that facilitates the accumulation of social capital among young, middle-aged, and elderly people is critical for both financial viability and social cohesion^4–6^.

The physical infrastructure of communities has long been considered to promote social interactions^7^. Indeed, micro-level evidence suggests that embeddedness to a physical structure enhances social capital^8^ in which the concept of embeddedness is defined as a situation where the individuals’ actions are refracted by the social relations within which they function^9^. The infrastructure physically glues each individual to a particular location where people are physically embedded within communities. Such a physical environment would help accumulate social capital as habitus^10^, habit^11^, or affordance^12^. However, these relationships, embedded in physical infrastructure, have seldom been subjected to rigorous empirical analysis. Moreover, most studies focus on projects in developed countries, with little about low- and middle-income countries^13^.

To bridge this lacuna in the existing literature, this study aims to discuss the impact of an innovative model called *Ibasho*, an elder-led community initiative that strives to strengthen social capital by bringing together community members to co-create physical infrastructure that is then managed by the elders. *Ibasho* literally means “a place where you can feel like yourself” in Japanese. This type of a place is often referred as “third place” in Europe^14^. The first *Ibasho* was established in 2013 in Ofunato, Japan^15^. Ofunato was among the areas most seriously affected by the Great East Japan Earthquake of 2011, and the project was conceived to serve as a place where older people could gather while recovering from the earthquake and contribute to the larger community. To preserve the local culture and maintain a sense of belonging, an old farmhouse was repurposed and redesigned to serve as a community hub. It became a place where older people shared their knowledge by conducting various activities, including cooking, gardening, festivals, etc. Despite being centered around elders, the community hub is open to everyone who wishes to stop by.

A group of local older people maintain operations in a self-sustaining manner. By empowering older people to engage in social activities rather than staying home alone, the project enhances their physical and mental health as well as social capital^16^. It was proven to be an effective intervention for post-disaster recovery in terms of easing the economic burden on the community, boosting resiliency in elders, and reducing social isolation along with economic, social, and health risks^17,18^. The building also serves as an evacuation center and provides elder-led disaster risk management planning.

The model was replicated in two other post-disaster areas—Ormoc in the Philippines and Matatirtha in Nepal—with support from the Global Facility for Disaster Reduction and Recovery (GFDRR) of World Bank. Ormoc and Matatirtha were affected by typhoon Yolanda in 2014 and the Gorkha earthquake in 2015, respectively, both of which seriously damaged the areas. The projects aimed to promote community preparedness and resilience by facilitating social capital among elders within their communities by replicating the Japanese experience. The *Ibasho*’s core principles were implemented through the unique empowerment process with local community members. To adapt the *Ibasho* model into different cultures, the project team conducted multiple workshops and co-created the program, operation, and the space over 18 months^19^. The present study performs a formal quantitative evaluation of replicability of the *Ibasho* model in the context of developing countries such as the Philippines and Nepal.

## Methods

### Sampling strategy

We conducted three rounds of surveys at each project site to assess the impact of *Ibasho* on physical and mental health and social capital. The project sites were Barangay Bagong Buhay in Ormoc in the Philippines and Ward 9 in Matatirtha in Nepal. Both sites are similar in size, making the results capable of being compared. The study protocol was approved by the Institutional Review Board of the University of Tokyo (approval No. 15-172, 16-35, 16-67). The details of the project are summarized in the project report^20^.

In the Philippines, the first round of the survey was implemented from October to December 2015. Before the survey, the initial pilot phase of the project had already started; some people had participated in the kick-off workshop and began vegetable gardening as a project activity. The sample was all the members of the Senior Citizens Association in the Barangay (village). The membership of the association includes all the residents above age 60. The survey was conducted in the elementary school with a door-to-door follow-up for those who did not appear. As a result, we covered 85.8% of the member list.

The second round of the survey was carried out door-to-door from October 2017 to May 2018, targeting all the respondents in the first round. However, we covered only 69% of those in the first round, because most of the others had died or moved. In addition, we added new respondents who had newly reached the age of 60. Between the first and the second survey, the elders opened a mobile café and repaired the community dining hall. We conducted the third round of the survey directly after the *Ibasho* building opened in January 2019. The sampling strategy was the same as in the first and the second rounds, and we were able to cover 75% of those who participated in the second round.

In Nepal, the first round of the survey was implemented from July to September 2016, after the kick-off workshop but before any other projects had started. We targeted all the people over 60 years of age living in Ward 9 and its neighboring wards: 8, 10, and 11. Unlike in the Philippines, however, there was no list of the elders. We collected data for 307 people with the assistance of the local coordinator. After the first round, the members started gardening vegetables, crafting earrings, and making a signboard to display the community’s evacuation map. The second survey was conducted from January to May 2018 following the same sampling strategy. We covered 77% of the samples in the previous round. After the second round, they started the reconstruction of the Chautari, a roadside rest spot beside a tree and now an *Ibasho* hub building. The third round of the survey was conducted from December 2018 to January 2019. In this round, we reduced the sample size by omitting respondents living outside of Ward 9 due to logistical challenges.

### Variables

As measures of mental and physical health, we employ the Kessler Psychological Distress Scale (K6) and Activities of Daily Living (ADL). K6 measures depression through a series of questions by asking how often the respondent had the following six feelings during the past 30 days: nervous, hopeless, restless and fidgety, so depressed that nothing could cheer him/her up, that everything was an effort, worthless (Question 11). The answers were on a 5-point Likert scale as follows: 0 = none of the time, 1 = a little of the time, 2 = some of the time, 3 = most of the time, and 4 = all of the time. ADL was calculated by asking whether the respondent could do the following self-care tasks: bathing, dressing, grooming, mouth care, toileting, transferring bed/chair, walking, climbing stairs, and eating (Question 12). The answers are coded by a 4-point Likert scale (1 = independent, 2 = needs help, 3 = dependent, 4 = cannot do) that was added up and then divided by the number of the items.

Since participation in *Ibasho* is expected to enhance social capital by connecting people within a community, we tested the impact of *Ibasho* on various outcomes related to social capital. The first is directly concerned with human relationships—the number of people to regularly talk with and the number of friends (both within the same village and block) were re-coded into a 5-point Likert scale: 0–9, 10–19, 20–29, 30–39, 40–49, and 50 or more (Questions 29–32). The survey also asked whether the respondents had friends or neighbors in the village besides their family whom they could ask for help (Question 33).

The second set of outcomes is psychosocial. The answers to the question of whether living in Barangay Bagong Buhay (village) or the City of Ormoc for the Philippines and the Gaon (village) and Ward 9 (municipality) for Nepal gives respondents a sense of community or a feeling of belonging are re-coded into 5-point Likert scale: very weak, weak, moderate, strong, and very strong (1–5) (Question 34). The survey also gauged the level of trust measured by the General Social Survey (GSS) by asking: Generally speaking, would you say that you can trust the following people? We then asked about people in the same block/village. The answers were re-coded into a 3-point Likert scale: 1 = they cannot be trusted, 2 = you can’t be too careful, and 3 = they can be trusted (Question 37). As a behavioral measure of social capital, the survey asked whether the respondents often leave the door unlocked (Question 38). The answers to a question asking whether the respondent feels that he/she is contributing to the village were re-coded into a 4-point Likert scale: 1 = no, 2 = small contribution, 3 = moderate, and 4 = significant contribution.

Covariates included age, gender, years of living in the community, marital status, working status, academic degree, monthly household expenditure (a 5-point Likert scale), family type, and housing situation. After dropping missing values in the control variables, the effective total sample size was 615 in the Philippines and 705 in Nepal. Summary statistics are shown in Tables S1 and S2 in the online appendix. Note that age, gender, number of years living in the community, and academic degree are dropped in the fixed effect models, as these variables are expected to be (virtually) perfectly correlated with individual and survey round fixed effects.

### Empirical strategy

In order to estimate the impact of *Ibasho* on physical and mental health and social capital, we employed a regression analysis. A potential threat to the impact evaluation is an issue around the endogeneity problem, i.e., that some unobservable characteristics might be correlated with participation in *Ibasho*. Thus, the estimated Ordinary Least Squares (OLS) coefficients should be interpreted as correlation rather than causality. Additionally, the longitudinal nature of the data enables us to estimate the same model with individual fixed effects (FE). In this model, we can arguably interpret the estimated coefficients as the impact of participation after controlling for time-invariant unobserved heterogeneities. Thus, by comparing the coefficients of OLS and FE, we can discuss what variables are associated with the participation status, as well as the impact on them, at least partly.

Another methodological issue is that some outcome variables are binary or ordinal. However, we estimate a linear regression model for several reasons^21^. Firstly, the magnitudes of coefficients are easy to interpret across equations. Secondly, we can easily incorporate fixed effects, which is the central aspect of our empirical strategy, by avoiding the classical incidental parameter problem^22^. Note that either OLS or ordered probit/logit models are known to have little difference in the qualitative results^23^.

### Approval for human experiments

The study protocol and data collection were carried out in accordance with the Institutional Review Board guidelines of the University of Tokyo (approval No. 15-172, 16-35, 16-67). All subjects are over 18, and informed consent was obtained from all subjects.

## Results

### Descriptive analysis

Table 1 provides the participation rates of *Ibasho* in each round for the Philippines and Nepal. The participation rate was high in the Philippines, and almost half of the respondents participated in the activities in the first round of the survey. The rate decreased to 40% in the second round, then increased to 58% in the third. This temporal fluctuation partly reflects the replacement of the sample due to death, moves, and new membership in the senior citizens’ association. In contrast, the participation rate remained 4–5% in each round for Nepal, suggesting low recognition of the activity within the community.

**Table 1:** The participation rate of *Ibasho.*

	Philippines		Nepal	
	Count	Mean	Count	Mean
Round 1	193	0.492	253	0.055
Round 2	192	0.401	283	0.042
Round 3	230	0.583	173	0.040

Authors’ calculations.

**Figure 1:**
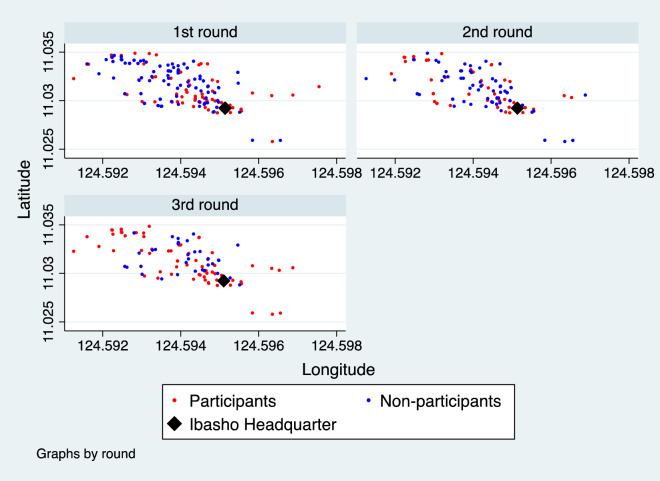
Household location and participation status in each round (Philippines). *Note*: The graph shows the location (latitude and longitude) of the sample households for participants (red) and non-participants (blue). It also shows the location of *Ibasho* headquarter (in black diamond).

Figure 1 shows the location of the sample households and participation status of the Philippines respondents. Most of the sample households are located within 1 km square, indicating high population density. In particular, people living near the headquarters tended to participate in the first and the second round, with participation becoming more widespread in the third round of the survey. Among the 81 people surveyed in all three rounds, 32% participated in *Ibasho* in all rounds, while 16% never participated. Due to data limitations, a similar graphical analysis cannot be applicable to Nepal. However, people living in the same village as the headquarters tended to participate more in the activities (*p* = 0.00). Therefore, even within a community, physical distance can impede participation in *Ibasho* activities.

### Regression analysis

**Figure 2:**
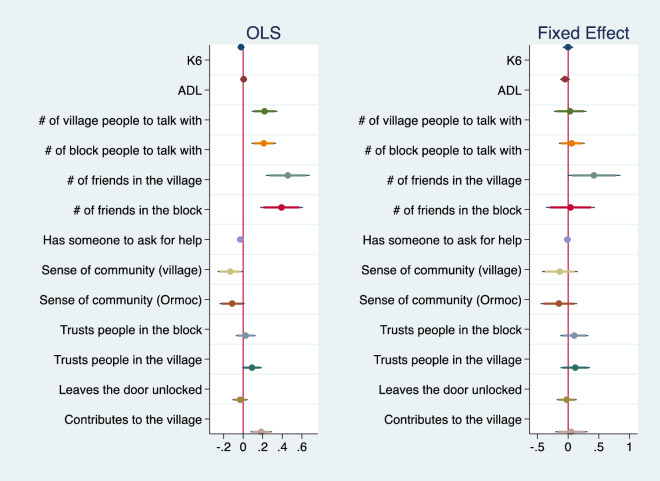
The impact of *Ibasho* participation on selected outcomes (Philippines). *Note*: The graph summarizes the coefficient on *Ibasho* participation for each outcome after controlling for age, gender, years of living in the community, marital status, working status, academic degree, monthly household expenditure, family type, and housing situation as well as round dummies.

Figure 2 summarizes the estimated coefficients on *Ibasho* from the OLS and fixed effect model for the Philippines. In the OLS model, participation in *Ibasho* is positively associated with number of friends and people to talk to on a daily basis. However, it is negatively associated with whether the respondents feel a sense of community toward the village and/or the city. After controlling for individual fixed effects, *Ibasho* participation affects only the number of friends in the village (*p* = 0.06). These comparisons suggest that people with many friends and acquaintances tend to participate in *Ibasho* and make friends through the activities. People who feel less of a sense of community probably participate in *Ibasho* because they want to feel more connected, but participation does not necessarily improve their sense of connection. The full estimation results are shown in Tables S3 and S4 in the online appendix. The propensity score matching for the pooled data using the same set of covariates also confirms that the qualitative results remain almost unchanged from the OLS results (Table S5).

**Figure 3:**
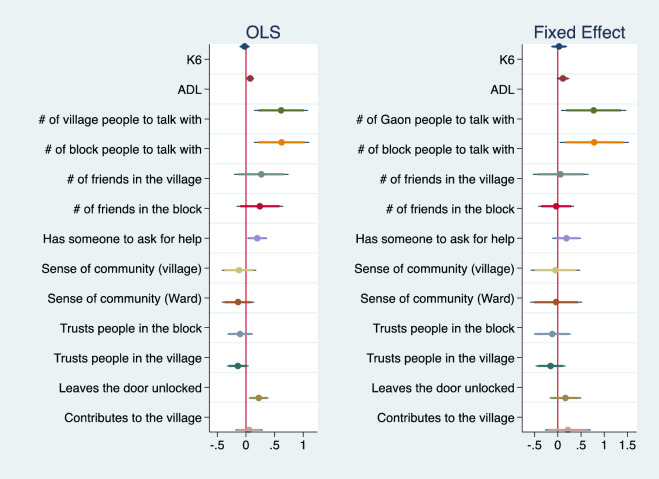
The impact of *Ibasho* participation on selected outcomes (Nepal). *Note*: The graph summarizes the coefficient on *Ibasho* participation for each outcome after controlling for age, gender, years of living in the community, marital status, working status, academic degree, monthly household expenditure, family type, and housing situation as well as round dummies.

Figure 3 summarizes the estimated coefficients on *Ibasho* from the OLS and fixed effect model for Nepal. A similar pattern can be confirmed in the case of Nepal. The number of people to talk to within village and block is positively correlated with *Ibasho* participation in the OLS model. This association remains statistically significant after controlling for unobserved time-invariant heterogeneities in the fixed effect model (*p* = 0.03 and 0.04, respectively). These results indicate that participating in *Ibasho* increased the number of acquaintances within a community. Those who participated also tended to have someone to ask for help and to leave the door unlocked in the OLS models. However, neither association was statistically significant in the fixed effect models. The full estimation results are shown in Tables S6 and S7 in the online appendix. The propensity score matching for the pooled data using the same set of covariates also confirms that the qualitative results remain almost unchanged from the OLS results (Table S8).

## Discussion

Increasing social capital is considered to be a key strategy for complementing the limited capacity of the public and private service delivery systems currently addressing challenges associated with an aging population. However, little is known about how to strengthen the role of the built environment in enhancing elders’ social capital, especially in developing countries. In aiming to fill this research gap, our study analyzed whether and how physical and social infrastructure enhanced social capital among elders in the Philippines and Nepal.

The overall regression results from the OLS and fixed effects models suggest that participation in *Ibasho* helped enhance social capital by increasing the number of friends within a village for the Philippines and the number of people to talk with within a village and block for Nepal. Since some of the estimated relationships become statistically insignificant with the inclusion of individual fixed effects to mitigate an estimation bias arising from unobserved time-invariant heterogeneities, there is an indication of reverse causality. People who already had an extensive network of friends and acquaintances in their community were more likely to participate in *Ibasho*. Yet, according to overall estimation results, we may conclude that the project contributed to enhancing social capital among elders in both cases.

Our results speak to the design of a community-based disaster risk management system. In the context of the Philippines, it has been argued that community mechanisms played an important role in escaping the devastation of Typhoon Yolanda, though a further risk communication system is necessary to empower citizens^24^. Other research also suggests that the centrality of communities is essential in the relocation process after Yolanda^25^. Although the communities considered in this study are different from these cases in that they were not subject to the community evacuation or relocation, incorporating *Ibasho* into the pre- and post-disaster management can be effective in reaching elders who are vulnerable to these risks.

It would also be useful to highlight the differences between these two communities. In the Philippines, participation in *Ibasho* increased the number of friends, or “strong ties,” rather than acquaintances, i.e., someone to talk to within a community, or “weak ties”^26^. This may indicate that the impact of *Ibasho* is on the intensive margin of human relationships: *Ibasho* strengthened weak ties, making them strong ties. In contrast, joining Nepal’s *Ibasho* broadened weak ties, its impact being realized along the extensive margin. This contrast may stem from the difference in the pre-existing social and built infrastructure in each community. A large proportion of the elders in the Philippines already knew each other through participation in the official senior citizens’ association, and there was no similar association in Nepal. In any case, further investigations of these issues require more detailed data on social network structure over time. These remain an important future research task.

Another important issue to be addressed in the future is the long-term impact of the project. The estimation results in our paper suggest that *Ibasho* enhanced intensive and extensive margins of human relationships in a relatively short-term period. However, all the results come from the data of the first phase of the project, covering a period of three years at most. How these relationships will change quantitatively and qualitatively in the long run remains unaddressed in the current study. Since that question directly concerns the sustainability of the project, further data collection and analysis are required.

## Supplementary Information

Supplementary Tables.

## Data Availability

All data needed to evaluate the conclusions in the study are described in the paper and/or the Supplementary Materials. While confidentiality concerns of our proprietary data prevent us from depositing our data in a public repository, the data sets used in this study will be made available upon request. Those requesting access to the data sets need to contact the corresponding author (Dr. Takeshi Aida) in writing (Takeshi_Aida@ide.go.jp).
