# Supplementary material for: Building social capital with elders’ leadership through a community hub “Ibasho” in the Philippines and Nepal
Source: Sci Rep. 2023 Mar 4;13:3652. doi: 10.1038/s41598-023-30724-7 (PMC9985593; doi:10.1038/s41598-023-30724-7)
Supplement: Supplementary file 1 — Supplementary Tables. [file 41598_2023_30724_MOESM1_ESM.docx]

**Building social capital with elders’ leadership through a community hub “*Ibasho*” in the Philippines and Nepal**

**Online Appendix**

| Takeshi Aida^*a^  *IDE-JETRO* | Emi Kiyota^b^  *National University of Singapore* | Yasuhiro Tanaka^c^  *Ibasho Japan* | Yasuyuki Sawada^d^  *University of Tokyo* |
| --- | --- | --- | --- |

**Affiliations:**

^a*^ Corresponding author: Research Fellow, Institute of Developing Economies, Japan External Trade Organization (IDE-JETRO). Wakaba 3-2-2, Mihama-ku, Chiba-shi, Chiba 261-8545, Japan. E-mail: [Takeshi_Aida@ide.go.jp](mailto:Takeshi_Aida@ide.go.jp)

^b^ Associate Professor, NUS Yong Loo Lin School of Medicine and College of Design and Engineering. 1E Kent Ridge Road, NUHS Tower Block Level 11, 119228 Singapore. E-mail: [ekiyota@nus.edu.sg](mailto:ekiyota@nus.edu.sg)

^c^ President, Ibasho Japan. 54-1, Tairabayashi, Massaki-Cho, Ofunato-City, Iwate 022-0001, Japan. E-mail: [yasuhiro@ibasho-japan.org](mailto:yasuhiro@ibasho-japan.org)

^d^ Professor, Faculty of Economics, University of Tokyo. 7-3-1 Hongo, Bunkyo-ku, Tokyo 113-0033, Japan. E-mail: [sawada@e.u-tokyo.ac.jp](mailto:sawada@e.u-tokyo.ac.jp)

**This PDF file includes:**

Table S1: Summary Statistics (Philippines)

Table S2: Summary Statistics (Nepal)

Table S3: Estimation Results from OLS Model (Philippines)

Table S4: Estimation Results from Fixed Effect Model (Philippines)

Table S5: Estimation Results from Propensity Score Matching (Philippines)

Table S6: Estimation Results from OLS Model (Nepal)

Table S7: Estimation Results from Fixed Effect Model (Nepal)

Table S8: Estimation Results from Propensity Score Matching (Nepal)

**Table S1:** Summary Statistics (Philippines)

| Philippines | 1st round | | | 2nd round | | | 3rd round | | |
| --- | --- | --- | --- | --- | --- | --- | --- | --- | --- |
| Dependent variables | count | mean | sd | count | mean | sd | count | mean | sd |
| K6 | 185 | 0.199 | 0.216 | 158 | 0.232 | 0.209 | 213 | 0.199 | 0.21 |
| ADL | 187 | 0.947 | 0.145 | 175 | 0.895 | 0.237 | 226 | 0.918 | 0.222 |
| # of village people to talk with | 188 | 1.191 | 0.642 | 177 | 1.209 | 0.688 | 223 | 1.363 | 0.899 |
| # of block people to talk with | 190 | 1.163 | 0.625 | 176 | 1.261 | 0.793 | 228 | 1.333 | 0.825 |
| # of friends in the village | 190 | 2.179 | 1.353 | 186 | 2.188 | 1.388 | 228 | 2.294 | 1.391 |
| # of friends in the block | 191 | 2.021 | 1.306 | 185 | 2.022 | 1.298 | 227 | 2.198 | 1.347 |
| Has someone to ask for help | 174 | 1.034 | 0.183 | 192 | 1.036 | 0.188 | 228 | 1.013 | 0.114 |
| Sense of community (village) | 193 | 1.689 | 0.852 | 185 | 1.978 | 0.897 | 224 | 2 | 0.734 |
| Sense of community (Ormoc) | 191 | 1.707 | 0.851 | 182 | 2.027 | 0.913 | 222 | 2.045 | 0.71 |
| Trusts people in the block | 183 | 2.344 | 0.551 | 167 | 2.353 | 0.612 | 205 | 2.634 | 0.54 |
| Trusts people in the village | 181 | 2.21 | 0.568 | 156 | 2.34 | 0.585 | 197 | 2.624 | 0.554 |
| Leaves the door unlocked | 192 | 0.271 | 0.446 | 177 | 0.367 | 0.483 | 219 | 0.388 | 0.488 |
| Contributes to the village | 190 | 2.463 | 0.71 | 184 | 2.402 | 0.645 | 227 | 2.48 | 0.674 |
| Independent variables | count | mean | sd | count | mean | sd | count | mean | sd |
| ibasho | 193 | 0.492 | 0.501 | 192 | 0.401 | 0.491 | 230 | 0.583 | 0.494 |
| Age | 193 | 68.596 | 6.562 | 192 | 68.505 | 6.15 | 230 | 68.509 | 6.354 |
| Female | 193 | 0.653 | 0.477 | 192 | 0.641 | 0.481 | 230 | 0.635 | 0.483 |
| # of years living in the community | 193 | 31.148 | 14.451 | 192 | 31.859 | 13.943 | 230 | 34.539 | 14.916 |
| Marital status: |  |  |  |  |  |  |  |  |  |
| Married but spouse is living elsewhere | 193 | 0.005 | 0.072 | 192 | 0.005 | 0.072 | 230 | 0.009 | 0.093 |
| Never married/Single | 193 | 0.187 | 0.391 | 192 | 0.161 | 0.369 | 230 | 0.065 | 0.247 |
| Widow/widower | 193 | 0.249 | 0.433 | 192 | 0.302 | 0.46 | 230 | 0.374 | 0.485 |
| Not married but living together | 193 | 0.016 | 0.124 | 192 | 0.021 | 0.143 | 230 | 0.009 | 0.093 |
| Married but spouse is living elsewhere | 193 | 0.041 | 0.2 | 192 | 0.042 | 0.2 | 230 | 0.057 | 0.231 |
| Currently working | 193 | 0.223 | 0.417 | 192 | 0.141 | 0.349 | 230 | 0.174 | 0.38 |
| Academic degree: |  |  |  |  |  |  |  |  |  |
| Middle school | 193 | 0.124 | 0.331 | 192 | 0.016 | 0.124 | 230 | 0.03 | 0.172 |
| High school | 193 | 0.233 | 0.424 | 192 | 0.234 | 0.425 | 230 | 0.196 | 0.398 |
| Bachelor | 193 | 0.176 | 0.382 | 192 | 0.203 | 0.403 | 230 | 0.196 | 0.398 |
| Vocational/Technical | 193 | 0.047 | 0.211 | 192 | 0.073 | 0.261 | 230 | 0.065 | 0.247 |
| Master/Doctoral | 193 | 0.005 | 0.072 | 192 | 0.031 | 0.174 | 230 | 0.017 | 0.131 |
| Monthly household expenditure | 193 | 2.663 | 1.7 | 192 | 2.568 | 1.72 | 230 | 2.174 | 1.615 |
| Family type: |  |  |  |  |  |  |  |  |  |
| Extended family | 193 | 0.192 | 0.395 | 192 | 0.354 | 0.48 | 230 | 0.326 | 0.47 |
| Single | 193 | 0.052 | 0.222 | 192 | 0.078 | 0.269 | 230 | 0.087 | 0.282 |
| Housing: |  |  |  |  |  |  |  |  |  |
| Temporary housing unit | 193 | 0.161 | 0.368 | 192 | 0.099 | 0.299 | 230 | 0.113 | 0.317 |
| Rental | 193 | 0.021 | 0.143 | 192 | 0.021 | 0.143 | 230 | 0.017 | 0.131 |
| Living at relative’s house | 193 | 0.031 | 0.174 | 192 | 0.057 | 0.233 | 230 | 0.057 | 0.231 |
| Other | 193 | 0 | 0 | 192 | 0.005 | 0.072 | 230 | 0 | 0 |

**Table S2:** Summary Statistics (Nepal)

| Nepal | 1^st^ round | | | 2^nd^ round | | | 3^rd^ round | | |
| --- | --- | --- | --- | --- | --- | --- | --- | --- | --- |
| Dependent variables | count | mean | sd | count | mean | sd | count | mean | sd |
| K6 | 243 | 0.680 | 0.269 | 280 | 0.816 | 0.194 | 168 | 0.842 | 0.175 |
| ADL | 242 | 0.909 | 0.200 | 283 | 0.865 | 0.288 | 173 | 0.890 | 0.261 |
| # of village people to talk with | 248 | 2.008 | 1.332 | 282 | 1.511 | 0.788 | 173 | 1.578 | 0.592 |
| # of block people to talk with | 245 | 1.955 | 1.291 | 282 | 1.429 | 0.709 | 173 | 1.474 | 0.556 |
| # of friends in the village | 247 | 1.891 | 1.193 | 282 | 1.457 | 0.769 | 173 | 1.572 | 0.725 |
| # of friends in the block | 248 | 1.569 | 0.941 | 282 | 1.429 | 0.723 | 173 | 1.590 | 0.723 |
| Has someone to ask for help | 238 | 0.803 | 0.399 | 281 | 0.484 | 0.501 | 173 | 0.642 | 0.481 |
| Sense of community (village) | 247 | 2.028 | 1.045 | 281 | 2.480 | 0.766 | 173 | 2.006 | 0.905 |
| Sense of community (Ward) | 246 | 2.264 | 1.038 | 281 | 2.552 | 0.755 | 173 | 2.006 | 0.931 |
| Trusts people in block | 236 | 2.530 | 0.661 | 228 | 2.399 | 0.525 | 137 | 2.927 | 0.261 |
| Trusts people in the village | 226 | 2.288 | 0.654 | 220 | 2.359 | 0.543 | 134 | 2.903 | 0.297 |
| Leaves the door unlocked | 243 | 0.276 | 0.448 | 277 | 0.108 | 0.311 | 163 | 0.319 | 0.468 |
| Contributes to the village | 244 | 2.500 | 0.873 | 281 | 1.843 | 0.809 | 172 | 1.924 | 0.802 |
| Independent variables | count | mean | sd | count | mean | sd | count | mean | sd |
| ibasho | 249 | 0.056 | 0.231 | 283 | 0.042 | 0.202 | 173 | 0.040 | 0.198 |
| Age | 249 | 70.783 | 8.141 | 283 | 72.163 | 8.588 | 173 | 77.040 | 61.874 |
| Female | 249 | 0.506 | 0.501 | 283 | 0.481 | 0.501 | 173 | 0.538 | 0.500 |
| # of years living in the community | 249 | 41.995 | 28.969 | 283 | 42.157 | 29.391 | 173 | 45.220 | 29.726 |
| Marital status: |  |  |  |  |  |  |  |  |  |
| Married but spouse is living elsewhere | 249 | 0.028 | 0.166 | 282 | 0.028 | 0.166 | 173 | 0.017 | 0.131 |
| Never married/Single | 249 | 0.008 | 0.089 | 282 | 0.011 | 0.103 | 173 | 0.012 | 0.107 |
| Widow/widower | 249 | 0.373 | 0.485 | 282 | 0.323 | 0.468 | 173 | 0.306 | 0.462 |
| Not married but living together | 249 | 0.008 | 0.089 | 282 | 0.007 | 0.084 | 173 | 0.012 | 0.107 |
| Currently working | 249 | 0.257 | 0.438 | 282 | 0.117 | 0.322 | 173 | 0.087 | 0.282 |
| Academic degree: |  |  |  |  |  |  |  |  |  |
| Lower secondary | 249 | 0.016 | 0.126 | 283 | 0.085 | 0.279 | 173 | 0.098 | 0.299 |
| Secondary | 249 | 0.044 | 0.206 | 283 | 0.049 | 0.217 | 173 | 0.029 | 0.168 |
| Higher secondary | 249 | 0.012 | 0.109 | 283 | 0.035 | 0.185 | 173 | 0.006 | 0.076 |
| Bachelor | 249 | 0.020 | 0.141 | 283 | 0.018 | 0.132 | 173 | 0.023 | 0.151 |
| Vocational/technical | 249 | 0.104 | 0.306 | 283 | 0.028 | 0.166 | 173 | 0.052 | 0.223 |
| Master/doctoral | 249 | 0.016 | 0.126 | 283 | 0.018 | 0.132 | 173 | 0.012 | 0.107 |
| No education | 249 | 0.667 | 0.472 | 283 | 0.633 | 0.483 | 173 | 0.642 | 0.481 |
| Monthly household expenditure | 249 | 5.361 | 1.069 | 283 | 5.300 | 2.515 | 171 | 5.906 | 2.221 |
| Family type: |  |  |  |  |  |  |  |  |  |
| Extended family | 249 | 0.205 | 0.404 | 283 | 0.025 | 0.156 | 173 | 0.064 | 0.245 |
| Single | 249 | 0.084 | 0.278 | 283 | 0.074 | 0.263 | 173 | 0.104 | 0.306 |
| Living with spouse | 249 | 0.040 | 0.197 | 283 | 0.035 | 0.185 | 173 | 0.029 | 0.168 |
| Housing: |  |  |  |  |  |  |  |  |  |
| Rental | 249 | 0.096 | 0.296 | 281 | 0.046 | 0.210 | 173 | 0.017 | 0.131 |
| Temporary housing unit | 249 | 0.096 | 0.296 | 281 | 0.007 | 0.084 | 173 | 0.017 | 0.131 |
| Living at relative’s house | 249 | 0.016 | 0.126 | 281 | 0.007 | 0.084 | 173 | 0.000 | 0.000 |
| Living at old age home | 249 | 0.032 | 0.177 | 281 | 0.046 | 0.210 | 173 | 0.058 | 0.234 |

**Table S3:** Estimation Results from OLS Model (Philippines)

**Table S4:** Estimation Results from Fixed Effect Model (Philippines)

**Table S5:** Estimation Results from Propensity Score Matching (Philippines)

|  | (1) | (2) | (3) | (4) | (5) | (6) | (7) | (8) | (9) | (10) | (11) | (12) | (13) |
| --- | --- | --- | --- | --- | --- | --- | --- | --- | --- | --- | --- | --- | --- |
| VARIABLES | K6 | ADL | # of village people to talk with | # of block people to talk with | # of friends in the village | # of friends in the block | Has someone to ask for help | Sense of community (village) | Sense of community (Ormoc) | Trusts people in the block | Trusts people in the village | Leaves the door unlocked | Contributes to the village |
|  |  |  |  |  |  |  |  |  |  |  |  |  |  |
| Ibasho | -0.022 | 0.010 | 0.172** | 0.189** | 0.546*** | 0.330*** | -0.044*** | -0.058 | -0.143** | 0.063 | 0.062 | -0.032 | 0.209*** |
|  | (0.021) | (0.022) | (0.072) | (0.083) | (0.131) | (0.117) | (0.015) | (0.083) | (0.071) | (0.056) | (0.058) | (0.043) | (0.063) |
|  |  |  |  |  |  |  |  |  |  |  |  |  |  |
| Observations | 556 | 588 | 588 | 594 | 604 | 603 | 594 | 602 | 595 | 555 | 534 | 588 | 601 |

Standard errors in parentheses *** p<0.01, ** p<0.05, * p<0.1

Age, gender, years of living in the community, marital status, working status, academic degree, monthly household expenditure, family type, housing situation, and round dummies are included in the calculation of the propensity score.

**Table S6:** Estimation Results from OLS Model (Nepal)

**Table S7:** Estimation Results from Fixed Effect Model (Nepal)

**Table S8:** Estimation Results from Propensity Score Matching (Nepal)

|  | (1) | (2) | (3) | (4) | (5) | (6) | (7) | (8) | (9) | (10) |
| --- | --- | --- | --- | --- | --- | --- | --- | --- | --- | --- |
| VARIABLES | K6 | ADL | # of village people to talk with | # of block people to talk with | # of friends in the village | # of friends in the block | Has someone to ask for help | Sense of community (village) | Leaves the door unlocked | Contributes to the village |
|  |  |  |  |  |  |  |  |  |  |  |
| Ibasho | -0.023 | 0.044* | 0.993*** | 1.046*** | 0.242 | 0.358 | 0.080 | -0.358*** | 0.334*** | 0.097 |
|  | (0.016) | (0.024) | (0.201) | (0.141) | (0.242) | (0.242) | (0.147) | (0.072) | (0.029) | (0.106) |
|  |  |  |  |  |  |  |  |  |  |  |
| Observations | 688 | 694 | 699 | 696 | 698 | 699 | 688 | 697 | 680 | 693 |

Standard errors in parentheses *** p<0.01, ** p<0.05, * p<0.1

Age, gender, years of living in the community, marital status, working status, academic degree, monthly household expenditure, family type, housing situation, and round dummies are included in the calculation of the propensity score. For the variables *sense of community (ward)*, *trusts people in the tole*, and *trusts people in the gaon*, the treatment overlap assumption has been violated, and the ATT was not estimated.
